# Sunitinib treatment for patients with clear-cell metastatic renal cell carcinoma: clinical outcomes and plasma angiogenesis markers

**DOI:** 10.1186/1471-2407-9-82

**Published:** 2009-03-12

**Authors:** Loukas F Kontovinis, Konstantinos T Papazisis, Panagiota Touplikioti, Charalambos Andreadis, Despoina Mouratidou, Alexandros H Kortsaris

**Affiliations:** 1 3rd Department of Medical Oncology, Theagenion Cancer Hospital, Al Simeonidi str. 2, 54007, Thessaloniki, Greece; 2 Applied Molecular Oncology Laboratory, Theagenion Cancer Hospital, Al Simeonidi str. 2, 54007, Thessaloniki, Greece; 3 Laboratory of biochemistry, Department of Medicine, Democritus University of Thrace, Alexandroupolis, Greece

## Abstract

**Background:**

Sunitinib is a protein tyrosine kinase-inhibitor targeting VEGFR, c-kit and PDGFR. It has been approved for the treatment of metastatic renal-cell carcinoma and gastrointestinal stromal tumors. Although it has been shown to prolong disease-free and overall survival in renal-cell carcinoma patients, only 70% of the treated population receive a clinical benefit (CB) from the treatment. Markers that could predict clinical benefit to sunitinib would be an important aid in monitoring and following their treatment. We assessed the outcome and plasma proangiogenic factors in patients with metastatic renal cell carcinoma (mRCC) treated with sunitinib in our institution.

**Methods:**

We have treated 42 patients with metastatic clear-cell renal carcinoma with sunitinib. Plasma concentrations of VEGF-A, sVEGFR2 and PDGF were determined by ELISA.

**Results:**

At the time of analysis 39 patients were evaluable for response and 30 patients had obtained a clinical benefit (CB). Median progression-free survival was 268 days (8.93 months) and median overall survival was 487 days (16.23 months). Interestingly, disease stabilization or objective response resulted in comparable overall survival. Most treatment-related adverse events were of mild-to-moderate intensity with one treatment-related death. Plasma sVEGFR2 and PDGF levels had no predictive value. Fold-increase in plasma VEGF was significantly lower in patients that obtained a CB as compared to patients that progressed after two cycles of treatment. Plasma VEGF did not increase in patients with initial CB at the time of progression.

**Conclusion:**

Sunitinib showed substantial activity in mRCC. Disease stabilization or objective response resulted in comparable overall survival and both outcomes should be considered positive. Fold-increase in plasma VEGF predicts for CB and could be a candidate marker. Progression after initial CB is not associated with elevated plasma VEGF, implying a different mechanism of resistance.

## Background

Clear-type renal cell carcinoma (RCC) represents 3% of all new cancer cases, 85% of all renal cancers and by far the most lethal urologic cancer. In 2008 it is estimated that there will be 54,390 new kidney and renal pelvis cancer cases (the majority of which are RCC) with a male to female ratio of 1.56:1 [1]. Renal cell carcinoma occurs more often in individuals aged 50 – 70 years old and it has been associated with several risk factors such as smoking, obesity and hypertension, although smoking probably is the most significant risk factor [2]. Renal cell carcinoma has been extremely resistant to chemotherapy, with disappointing response rates (around 6%) [3]. The only effective treatment until recently was immunotherapy with interferon-α and interleukin-2 with higher response rates around 10–15% [4,5].

The majority of RCC occurs sporadically but there is a small percentage of 1 – 4% that appears to carry a genetic predisposition [6]. Both sporadic and inherited clear type RCC is strongly associated with mutations in Von Hippel Lindau (VHL) tumor suppressor gene [7]. VHL gene is located on chromosome 3 and has a key role in the hypoxia inducible pathway, inducing hypoxia inducible factor (HIF-1 alpha and beta [8]) ubiquitinosis in the presence of oxygen. HIF-1α is stable in hypoxia, but in the presence of oxygen it is targeted for proteasomal degradation by the ubiquitination complex VHL [9]. HIF is a transcriptional complex that mediates the response of human cells to hypoxic environment resulting in the transcription of genes as vascular endothelial growth factor (VEGF), platelet-derived growth factor (PDGF), transforming growth factor-α (TGF-α) and erythropoietin [10]. Platelet derived growth factor receptors (PDGFRs) and vascular endothelial growth factor receptors (VEGFRs) play an essential role in tumor angiogenesis and growth [11]. VHL – HIF-1 – VEGF pathway is therefore deregulated in RCC and it represents a reasonable therapeutic target for renal cell carcinoma [12].

Sunitinib malate (SUTENT^®^, SU11248; Pfizer Inc; New York, USA) is an oral multitargeted tyrosine kinase inhibitor of VEGFR-1, VEGFR-2, Fms-like tyrosine kinase receptor 3 (FLT3), c-KIT (stem-cell factor [SCF] receptor) and PDGFR [13,14]. Phase I trials established the safety of 50 mg/day sunitinib (4 weeks on, 2 weeks off) and showed responses in a variety of tumors including RCC and gastrointestinal stromal tumors (GIST) [15]. Phase II trials in cytokine-resistant RCC showed a remarkable efficacy with a disease control rate of 65% and a median time-to-progression of 8.7 months [16]. A large randomized phase III trial comparing sunitinib to interferon-α resulted in statistically significant higher objective response rates (31% vs. 6%, P < 0.001) and a longer progression-free survival (11 vs. 5 months), with a hazard ratio of 0.42 (0.32 to 0.54, P < 0.001) [17]. Sunitinib is already approved for the treatment of metastatic RCC and GIST and clinical trials are ongoing in other indications as well as in the adjuvant RCC treatment.

The majority of sunitinib-treated patients obtain a clinical benefit in the form of either objective response or disease stabilization (31% and 48% respectively in the phase III trial). Furthermore there are patients who obtain a clinical benefit that do not show a response in the beginning of treatment and respond later in the course of treatment (unpublished observation). It is therefore important to recognize this population as RECIST criteria seem to be of less value [18,19] and PFS is not directly related to response rates, at least in the case of targeted treatments [20]. Soluble forms of pro-angiogenic growth factors such as VEGF-A and receptor sVEGFR2 or PlGF can be measured in the plasma of patients by ELISA and used as surrogate markers for response [21]. In the case of sunitinib treatment for metastatic RCC it has been shown that plasma VEGF levels increase after treatment and the ratio of post-treatment VEGF to the pre-treatment levels is different in patients that respond vs. patients with stable disease or disease progression [22].

We have evaluated 42 patients with metastatic clear-type renal cell carcinoma that were treated in our department with sunitinib (between June 2006 and August 2008) and their plasma levels of proangiogenic markers. There was a different pattern of VEGF level responses in patients with sunitinib-refractory disease (patients who experienced a disease progression after the first two cycles of treatment) and patients with sunitinib resistance (patients who originally obtained a clinical benefit and later progressed while on treatment). This may have implications in the treatment of these two different patient groups.

## Methods

### Eligibility criteria

Patients with metastatic clear type RCC, an Eastern Cooperative Oncology Group performance status ≤ 3 and age ≤ 75. Patients could be enrolled either as first line metastatic or second line after failure of cytokine treatment. All patients had an appropriate renal (Cr ≤ 2), liver (transaminases ≤ 3 times the maximum limit of normal values, total bilirubin ≤ 2 times the max limit of normal values) and bone marrow function (Hb ≥ 10.0, WBC ≥ 3000, NEU ≥ 1000, and platelets ≥ 100,000). Patients had not severe cardiologic disease and were required to have a recent echocardiogram with left ventricular ejection fraction at least 50%. In patients with a LVEF around 50% a MUGA test was performed.

The study was approved by the "Theagenion" Cancer Hospital ethics review board and was undertaken in accordance with the Declaration of Helsinki and Good Clinical Practice Guidelines. All patients were informed on their participation in this trial and signed the appropriate consent form.

### Drug administration

Sunitinib was administrated in the usual scheme (four weeks on treatment, followed by two weeks off treatment, on a six – week cycle). Starting dose was 50 mg daily and in case of intolerance there was a dose reduction to 37.5 mg daily. No further dose reduction was needed in the study.

### Examinations on treatment

Physical examination, ECOG performance status, CBC with differential and platelet count, complete biochemical profile at every scheduled visit on day 0, 15, 30, 45, 60, 75, 90 and at the beginning and the end of every subsequent treatment cycle. Thyroid function was accessed periodically. Toxicity was evaluated using National Cancer Institute Common Toxicity Criteria version 3.0. Primary and metastatic disease was assessed either by computed tomography scan or magnetic resonance imaging scan before starting the treatment and at the end of every two cycles (three months on treatment). RECIST criteria were used for response evaluation [23].

### Bioanalytics

Blood samples (15 ml) were collected from every patient and centrifuged (1500 rpm for 5 minutes) to separate plasma; aliquots were stored at -80°C and thawed only once or twice. Plasma concentration of VEGF-A, PDGF-AB and soluble VEGFR-2 were determined by enzyme-linked immunosorbent assay (ELISA) according to the manufacturer's instructions (R & D Systems).

### Post-sunitinib treatment

Patients who progressed on sunitinib and had a performance status at least 2, were treated with second (or third) line sorafenib. One patient that progressed on sorafenib but still remained in an eligible performance status is currently treated with temsirolimus.

### Data analysis

Protein plasma concentration data and correlations with response were analyzed with Microsoft Excel. Comparison results from Student's t-test with a p less than 0.05 were considered statistically significant. Kaplan-Meyer and log-rank tests were performed using GraphPad Prism 5 for Windows.

## Results

### Patient Characteristics

We have examined 42 patients with clear-cell metastatic carcinoma (31 men and 11 women) that received 50 mg of sunitinib daily for 30 out of 45 days per cycle (median age 64, range 25 – 75). Sunitinib was given either as first line treatment (n = 29) or as second line after failure of IFN-α (n = 13). Survival data were obtained from 40 patients. All patient characteristics are summarized in tables 1 and 2.

**Table 1:** Patient characteristics

Patient characteristics		%
**Number**		
Total included	42	100
Assessable for response	39	93

**Sex**		
Male	31	74
Female	11	26

**Age (years)**		
Median	64	
Range	25–75	

**Performance status**		
0	18	43
1	17	41
2	7	17

**MSKCC risk classification**		
Favorable	3	7
Intermediate	23	55
Poor	16	38

**Previous treatments**		
Nephrectomy	35	83
Interferon-α	13	31
Interferon-α + chemotherapy	6	14
Radiotherapy	14*	33

* Radiotherapy was directed against the primary tumor bed (as "adjuvant") in three patients; the rest (11 patients) received radiotherapy to the metastatic site (bone)

**Table 2:** Sites of relapse or metastasis

Site	Number	%
Local relapse	5	12
Bone*	17	41
Liver	4	10
Lung	34	81
Lymph nodes	8	19
Brain	1	2
Other sites	3	7

*25 patients (60%) with at least 2 metastatic sites*		

* All patients with metastatic bone disease received bisphosphonate treatment (ibadronate or zoledronate). Eleven patients needed radiotherapy to the metastatic bone disease and three required an operation as well.

### Response to treatment (table 3)

**Table 3:** Objective responses after two cycles of treatment in all evaluable patients (n = 39) and in the intention-to-treat population (n = 42).

Response	Number	% (evaluable pts)	% (ITT)
Total number of evaluable patients	39		
Objective responses	19	49	45
Complete response	0	0	0
Partial response	19	49	45
Disease stabilization	11	28	26
Disease progression	9	23	21

From the 42 patients that were enrolled in the study 39 were evaluable for response at the time of analysis; 30 patients (77% in evaluable patients and 71% in the intention-to-treat [ITT] population) had a clinical benefit (remission or disease stabilization). One patient received less than two cycles because he developed a severe reaction to sunitinib and he was switched to sorafenib and another died from pulmonary embolism (reported as severe adverse event) at cycle 2. From the 30 patients that had a clinical benefit, 19 patients (49% in evaluable patients and 45% in the ITT population) had a partial response to treatment whilst 11 (28% in evaluable patients and 26% in the ITT population) of them obtained a disease stabilization. Nine patients (23% in evaluable patients and 21% in the ITT population) had disease progression and treatment was discontinued. The 42 patients that were enrolled in our study received an average of 6 cycles of sunitinib (range 1 – 13). Two patients experienced disease flare up during the off-treatment periods and continued a non stop treatment with 37.5 mg of sunitinib daily.

**Figure 1:**
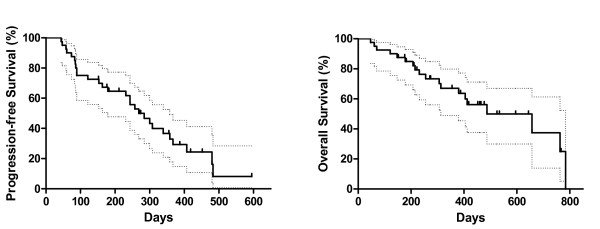
**Progression-free (PFS) and overall survival (OS) of metastatic RCC patients treated with sunitinib**. Dotted lines represent confidence intervals. Median PFS = 268 days (8.9 months, 95% CI: 195 to 342 days) and median OS = 487 days (16.2 months, 95% CI, 236 to 738 days).

**Figure 2:**
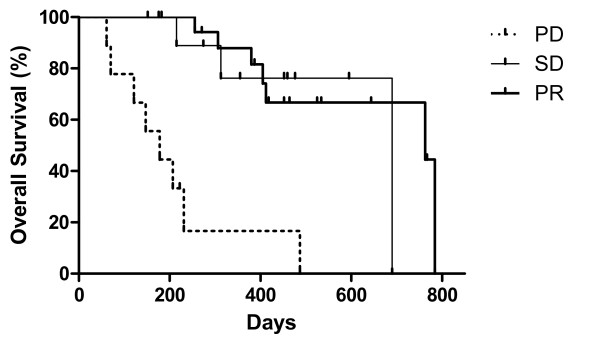
**Overall survival by type of response on first evaluation (after 2 cycles of sunitinib treatment)**. Median OS for patients with progressive disease (PD) = 178 days (n = 9), for stable disease (SD) = 657 days (n = 11) and for partial response (PR) = 763 (n = 19). There is no statistical difference between SD and PR patients (p = 0.6883 with log-rank test) but there was a statistical significant difference between the PD and PR group (p < 0.0001, log-rank test) or between the PD and SD group (p = 0.0008).

Median progression free survival was 268 days (Kaplan – Meyer, 95%, CI: 195 – 342 days) whereas median overall survival was 487 days (95% CI, 236 – 738 days) (figure 1). Overall survival was longer in patients that obtained a clinical benefit than in patients that exhibited a disease progression on first evaluation, after two cycles of sunitinib treatment (763 vs. 178 days, p < 0.0001, log-rank test). Interestingly, there was not any difference in overall survival between patients that showed disease stabilization or objective response on first evaluation (p = 0.6883, log-rank test, figure 2).

### Adverse events

Most important adverse events are summarized in table 4; the majority of them were grade 1 or 2. Most frequent event was fatigue that appeared in 24 patients (57%) and usually from day 15 until day 30 of each cycle. Although it was a symptom that impeded most of the patients to work regularly, it was almost fully reversible during the off treatment period.

**Table 4:** Adverse events

Adverse event	Any grade	%	Grade 3	%
Yellow skin discoloration	25	59	-	-
Fatigue	24	57	1	2
Taste changes	22	52	-	-
Mucositits	21	50	0	0
Hypertension	19	45	7	17
Epigastralgia	15	36	0	0
Nausea/vomiting	14	33	0	0
Anorexia	12	29	2	7
Diarrhea	11	26	0	0
Facial edema	9	21	1	2
Hand and foot syndrome	6	14	0	0
Thyroid function abnormalities	5	12	0	0
Thrombopenia	5	12	1	2
Anemia	4	10	1	2
LVEF reduction*	3	7	1	2
Myalgia	3	7	0	0
Neutropenia	3	7	1	2
Constipation	2	7	0	0
Pneumothorax	1	2	1	2
Hemoptysis	2	7	0	0

There was one grade 5 adverse event (a patient who developed a massive pulmonary embolism, see text).

* Any grade: Reductions in LVEF more than 15% from baseline or LVEF less than 50%. Grade 3: 30–40%.

Hypertension (any grade) was presented in 19 patients (45%), usually during the first two cycles of treatment and it was treated successfully with common antihypertensive medications. There was not any correlation between hypertension and response, progression-free survival or overall survival (data not shown). Seven patients developed grade 3 hypertension (required more than one drug for control of blood pressure).

Hematological toxicity presented in 7 patients (17%) with neutropenia, thrombocytopenia and anemia in 3 (7%), 5 (12%) and 4 (10%) patients respectively. Anemia was treated with erythropoietin-B whilst neutropenia resolved spontaneously and did not require treatment. There was only one event of pancytopenia (grade 4 febrile neutropenia, grade 3 thrombocytopenia and grade 3 anemia) after only two weeks of treatment. Sunitinib was discontinued and the patient went on to receive sorafenib with a stabilization of disease for 9 months.

Skin toxicity with yellow skin discoloration appeared in 25 patients (60%), while six (14%) of them developed additionally hand and foot syndrome with painful bullous lesions. Mucositis (principally stomatitis) was frequently reported (50%), as well as facial edema (21%) which was mostly located around the eyelids. There was one patient who presented with a generalized body edema and was hospitalized until resolution. She refused further treatment with sunitinib and was treated with sorafenib.

Other less frequent but equally important adverse events were anorexia (29%), taste changes (52%), nausea/vomiting (33%), epigastralgia (36%), diarrhea (26%) and thyroid function abnormalities (12%).

We observed only one fatal adverse event on a patient who underwent a massive pulmonary embolism. The patient had no history of deep vein thrombosis or other hypercoagulative disease.

A seventy years old woman developed a spontaneous pneumotorax proved to be provoked by remission of sub-pleural metastatic foci. She was surgically treated and sunitinib was continued until disease progression.

Three patients missed one cycle of treatment due to a LVEF reduction without symptoms or signs of heart failure. The minimum observed LVEF was 30%. Normal cardiac function was restored after a temporary break of treatment.

### Plasma angiogenesis markers

Evaluation of clinical response was performed every two cycles of treatment and evaluation of plasma biomarkers was performed every 15 days for the first 2 cycles, then at beginning and end of treatment cycles subsequently. Although all time points were analyzed, we present the data for the beginning and the end of each cycle (days 0 and 30, 45 and 75, etc). The plasma levels of all markers in the middle of each cycle (e.g. day 15) in general followed the trend between the start and the end of the cycle. The number of patients that started each cycle is 39, (cycles 1 and 2), 29 (cycle 3), 28 (cycle 4), 25 (cycle 5) and 23 (cycle 6). Baseline levels of all markers are presented on additional file 1.

For a better analysis patients were grouped according to disease response after the first two cycles. A "clinical benefit" group included both responders and patients that achieved disease stabilization whereas patients in the other group experienced progression of the disease.

**Figure 3:**
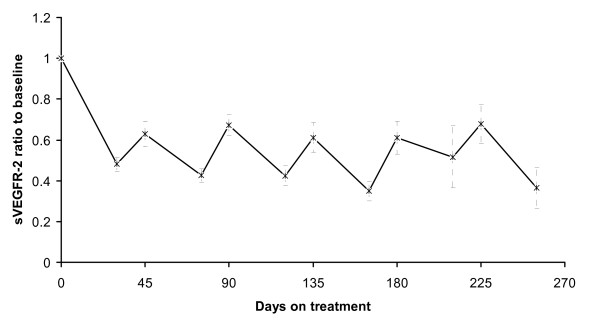
**Plasma sVEGFR2 ratio to baseline in metastatic RCC patients treated with sunitinib**. Vertical error bars represent SEM. Days 0, 45, 90, 135, 180 and 225 represent the beginning of cycles 1, 2, 3, 4, 5 and 6, respectively.

Baseline sVEGFR-2 levels were comparable in both groups at all time points. sVEGFR2 was decreased during sunitinib treatment and increased during the off-treatment periods at a similar manner as at has been observed by other groups as well (figure 3).

**Figure 4:**
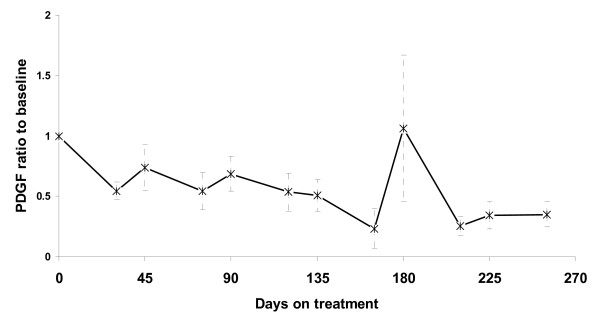
**Plasma PDGF levels in metastatic RCC patients treated with sunitinib (ratio to baseline values)**. Vertical error bars represent SEM. Days 0, 45, 90, 135, 180 and 225 represent the beginning of cycles 1, 2, 3, 4, 5 and 6, respectively.

Treatment with sunitinib lowered plasma PDGF levels in both subgroups. Baseline plasma PDGF was lower in the clinical benefit group (mean 16 ng/ml vs. 25 ng/ml, p = 0.15) and remained lower throughout the whole treatment period. However this was never statistical significant nor could have a predictive value. PDGF levels displayed a similar fluctuation during treatment cycles as sVEGFR-2 (figure 4).

**Figure 5:**
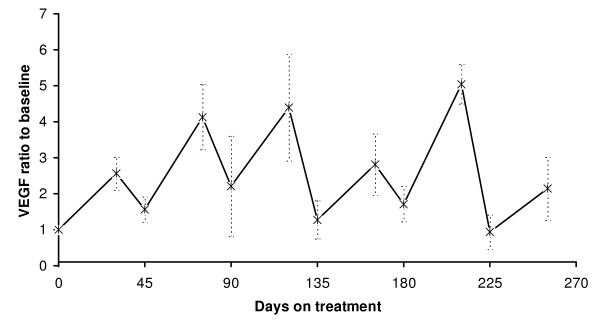
**Plasma VEGF-A levels in metastatic RCC patients treated with sunitinib (ratio to baseline values)**. Vertical error bars represent SEM. Days 0, 45, 90, 135, 180 and 225 represent the beginning of cycles 1, 2, 3, 4, 5 and 6, respectively.

**Figure 6:**
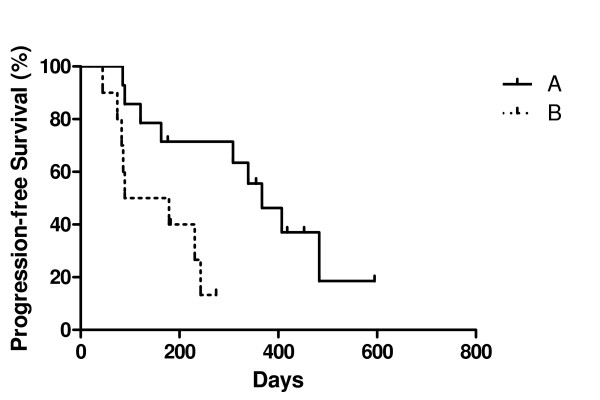
**Progression-free survival by fold increase in plasma VEGF-A**. Group A (n = 10): Patients that had lower than average fold increase of plasma VEGF-A at the end of cycle 2, Group B (n-14): Patients that had higher than average fold increase of plasma VEGF-A at the end of cycle 2. Median PFS = 134 vs. 367 days, p = 0.010 by log-rank test, HR = 0.2 (95% CI = 0.059–0.68).

**Figure 7:**
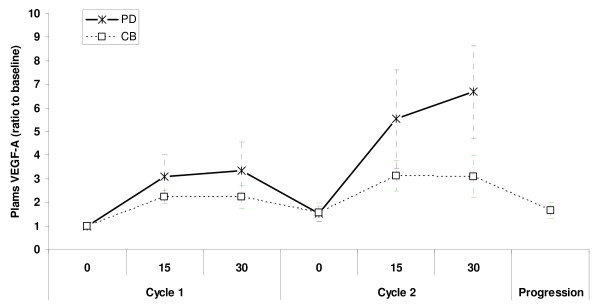
**Changes in plasma VEGF-A (ratio to baseline VEGF-A) according to clinical benefit during the first two treatment cycles and at the time of later progression (for patients that initially obtained a clinical benefit)**. CB: Clinical benefit; PD: Disease progression

Baseline plasma VEGF-A levels were almost identical in the clinical benefit vs. the non responders group (290 and 270 pg/ml respectively). Sunitinib treatment increased plasma VEGF-A in both groups (figure 5). However this was much higher in the group of patients who experienced a disease progression after the first two cycles of treatment (mean fold increase of 6.7), compared to the clinical benefit group (mean fold increase of 3.1, p = 0.033), resulting in significantly higher plasma VEGF-A levels at the end of the first two cycles in the non responders group (109 ng/ml versus 54 ng/ml, p = 0.010). The group of patients that had a higher than average fold increase in plasma VEGF-A by the end of cycle 2, had a statistically significant lower progression-free survival compared to patients with smaller increases in VEGF-A (median PFS 134 vs. 367 days, p = 0.010, HR = 0.2 [95% CI = 0.059–0.68], figure 6). At the time of progression patients that initially obtained a clinical benefit from sunitinib treatment did not increase plasma VEGF-A levels (as patients in the progression group) (figure 7), measured at the end of the last treatment cycle.

## Discussion

We have treated 42 metastatic renal cell carcinoma patients with sunitinib 50 mg per day, 4 weeks on and two weeks off. Our results are in concert with other reports, showing comparable clinical benefit ratio and a similar time to progression. In the phase II trial reported by Motzer [16] sunitinib treatment resulted in a clinical benefit for the majority of patients (67%) and a median time-to-progression of 8.7 months. The larger, phase III trial (sunitinib vs. interferon-alpha) exhibited a progression-free survival of 11 months in the sunitinib arm [17]. We report a progression-free survival of 8.9 months and an overall survival of 16 months. An interesting observation is that patients who obtained disease stabilization after two cycles of treatment had the same PFS and OS with patients that showed disease regression with the RECIST criteria. This implies that the aim of treatment is not to obtain an objective response but rather to achieve a clinical benefit by inhibiting the progression of the disease.

Overall, sunitinib treatment was tolerated well with the majority of patients reporting grade I-II side effects. Fatigue and taste changes were the most common side effects that impaired patients' quality of life. Taste changes particularly appeared after several cycles of treatment and persisted as a problem until discontinuation of sunitinib. However there was not any significant weight loss in this cohort of patients, even though some of them experienced stomatitis and gastric discomfort as well. Hypertension was a common problem, but contrary to what reported by Rixe et al [24], it did not predict for treatment effectiveness in our patients.

Plasma angiogenesis markers were evaluated in all patients every two weeks of treatment during the first two cycles and on the start and end of treatment cycles thereafter. Plasma sVEGFR-2 and PDGF levels fluctuated during the treatment period at a similar fashion as have been reported by other groups as well. However, there was not any predictive value in either of these two markers.

Plasma VEGF-A levels increased after treatment and fluctuated during the on-off treatment periods in a similar way as observed by other groups. VEGF-A levels increased at the end of cycle 2 and this was more prominent in patients that had disease progression than in patients that obtained a clinical benefit. DePrimo et al reported on 63 patients with metastatic RCC treated with sunitinib after failure of first-line cytokine therapy. They showed an increase in plasma VEGF which was more prominent in the patients with a partial response (PR) than in the non-PR group [22]. We have observed completely the opposite, though analyzing the results based on a different grouping of patients. Since disease stabilization is considered a positive outcome in patients treated with targeted therapies, we grouped patients in two groups. Group 1 consisted of patients who obtained a clinical benefit (complete response, partial response or stable disease) and remained on treatment. Group 2 was formed from patients with progressive disease (according to RECIST) that discontinued treatment after two full cycles of sunitinib. Patients with clinical benefit had a tendency to increase VEGF-A levels at a much lower fold ratio than patients with disease progression. On the contrary, when patients from the clinical benefit group experienced a secondary progression (resistance to treatment) they did not increase plasma VEGF-A. This may imply a different mechanism between primary (disease refractory to treatment) and secondary resistance. We hypothesize that patients with disease refractory to treatment may benefit from an additional anti-VEGF treatment like Bevacizumab [25] (though bevacizumab/sunitinib combination trials have recently been halted due to cases of microangiopathic hemolytic anemia [26]). This may not be the case for the secondary resistance where other factors may contribute to sunitinib failure. On the other hand, sunitinib has a substantial clinical activity after bevacizumab failure, implying a different mechanism of resistance [21].

## Conclusion

In conclusion, sunitinib showed a substantial antimumor activity in patients with metastatic renal cell carcinoma. The toxicity profile was favorable, with a few severe adverse events. Obtaining a clinical benefit, either with disease stabilization or with reduction of tumor burden resulted in comparable overall survival and both outcomes should be considered positive. The fold-increase in plasma VEGF levels predicted for clinical benefit and overall survival and could be a candidate marker if confirmed in larger series. On the other hand, progression after initial clinical benefit from sunitinib was not associated with elevated plasma VEGF levels, implying a different mechanism of resistance.

## Competing interests

The authors declare that they have no competing interests.

## Authors' contributions

LK was responsible for the treatment of the patients, the collection of the data and biologic samples as well as the ELISA tests and the preparation of the manuscript. KTP designed the study and participated in the evaluation of the data and the writing of the manuscript. PT collected the data and helped with the plasma databank. CA participated in the clinical part of the study. DM supervised the project and evaluated the patients. AHK overviewed the analysis of the data and the manuscript.

All authors have read and approved the present manuscript.

## Pre-publication history

The pre-publication history for this paper can be accessed here:

http://www.biomedcentral.com/1471-2407/9/82/prepub

## Supplementary Material

Additional file 1 Mean plasma values of angiogenesis markers in patients that progressed (PD) or had a clinical benefit (CB) on evaluation after two cycles of treatment. Click here for file
