# Supplementary material for: Sunitinib treatment for patients with clear-cell metastatic renal cell carcinoma: clinical outcomes and plasma angiogenesis markers
Source: BMC Cancer. 2009 Mar 12;9:82. doi: 10.1186/1471-2407-9-82 (PMC2662874; doi:10.1186/1471-2407-9-82)
Supplement: Additional file 1 — Mean plasma values of angiogenesis markers in patients that progressed (PD) or had a clinical benefit (CB) on evaluation after two cycles of treatment. [file 1471-2407-9-82-S1.doc]

**Additional file 1**

Mean plasma values of angiogenesis markers in patients that progressed (PD) or had a clinical benefit (CB) on evaluation after two cycles of treatment.

|  |  | **Cycle 1** |  | **Cycle 2** |  | **Cycle 3** |  | **Cycle 4** |  | **Cycle 5** |  | **Cycle 6** |  |
| --- | --- | --- | --- | --- | --- | --- | --- | --- | --- | --- | --- | --- | --- |
|  |  | Day 0 | Day 30 | Day 0 | Day 30 | Day 0 | Day 30 | Day 0 | Day 30 | Day 0 | Day 30 | Day 0 | Day 30 |
| **sVEGFR2 (ng/ml)** | **PD** | 9.51 | 4.40 | 4.70 | 2.68 |  |  |  |  |  |  |  |  |
|  | **CB** | 9.04 | 4.00 | 5.51 | 3.81 | 5.82 | 3.64 | 4.93 | 2.92 | 5.04 | 3.69 | 6.06 | 3.49 |
| **PDGF (ng/ml)** | **PD** | 24.78 | 13.83 | 16.11 | 15.05 |  |  |  |  |  |  |  |  |
|  | **CB** | 15.96 | 7.82 | 7.78 | 5.20 | 7.78 | 9.17 | 6.83 | 3.11 | 6.96 | 4.50 | 5.52 | 2.34 |
| **VEGF (ng/ml)** | **PD** | 0.27 | 0.66 | 0.35 | 1.09 |  |  |  |  |  |  |  |  |
|  | **CB** | 0.29 | 0.49 | 0.28 | 0.54 | 0.21 | 0.50 | 0.29 | 0.53 | 0.32 | 0.50 | 0.24 | 0.53 |
